# Do teachers differ in terms of their empathy toward liked students and disliked students? The role of empathic motivation

**DOI:** 10.3389/fpsyg.2025.1570187

**Published:** 2025-04-30

**Authors:** Xia Wang, Chenyu Shuangguan, Yuesheng Huang

**Affiliations:** ^1^School of Education, Hunan First Normal University, Changsha, Hunan, China; ^2^College of Education Science and Technology, Nanjing University of Posts and Telecommunications, Nanjing, China

**Keywords:** teacher-perceived student likability, teacher empathy, empathic motivation, anticipated emotional exhaustion, mediating effect

## Abstract

**Introduction:**

Teacher empathy has been proven to be highly relevant to both the educational process and outcomes. Therefore, exploring its influencing factors and developing effective cultivation strategies are highly importance. The present study aimed to examine the effects of teacher-perceived student likability on teacher empathy and to further explore the role of empathy motivations.

**Methods:**

In Study 1, 138 primary and secondary school teachers (mean age = 38.0 ± 8.8 years) reported their anticipated emotional exhaustion, empathic motivation, and empathic reaction when they read a text that described a negative event involving either a disliked or liked student. In Study 2, another 221 primary and middle school teachers (mean age = 34.8 ± 10.1 years) took part in an intervention designed to activate empathic motivation.

**Results:**

The results of study 1 showed that teachers felt less empathy in the former context. In addition, anticipated emotional exhaustion and empathic motivation serially mediated the effect of teacher-perceived student likability on teacher empathy. The results of Study 2 showed that teachers' empathy toward disliked students improved and that the likable empathy bias was eliminated when empathic motivation was primed.

**Discussion:**

These findings suggest that empathic motivation plays a crucial role in likable empathy bias among teachers in that it not only functions as a key mechanism underlying this bias but also emerges as a potential pathway for mitigating such bias. Our research has important theoretical and practical significance.

## 1 Introduction

Empathy, which refers to the ability to understand and share others' emotional experiences, is a common interpersonal phenomenon in human society (Weisz and Cikara, 2021). This ability enables individuals to perceive others' emotions and understand their behaviors quickly and therefore contributes to the establishment and maintenance of interpersonal relationships (Kim et al., 2022) as well as the generation of prosocial behavior (Fu et al., 2022). In flied of education, teaching is not merely the transmission of knowledge but also an inherently relational and emotional practice. Beyond instructional responsibilities, teachers play a crucial role in shaping students' social-emotional development and fostering an inclusive learning atmosphere (Stojiljković et al., 2012; Watt et al., 2021). To fulfill these roles effectively, teachers must engage in emotional labor, requiring them to recognize and respond to students' perspectives and emotions with empathy. Teacher empathy, as a critical aspect of professional teaching competence, enables educators to attune to students' needs and establish meaningful connections (Aldrup et al., 2022; Meyers et al., 2019). Recent studies highlight that an empathetic teaching approach contributes to a more supportive classroom climate, enhances student motivation, and facilitates deeper engagement in learning activities (Wink and Smith, 2021; Zhang, 2022; Cai et al., 2023). Furthermore, the ability to empathize with students is not only beneficial for learners but also fosters teachers' own professional fulfillment and resilience in the face of pedagogical challenges (Zhu et al., 2019; Wink and Smith, 2021). Given the importance of teacher empathy in educational practice, investigating the factors influencing this phenomenon and ways of cultivating teacher empathy is imperative.

In teaching practice, teachers often show differing levels of preference toward their students (Chang et al., 2004, 2007; Mercer and DeRosier, 2008). For example, teachers tend to dislikes students who are that disrespectfulness, have poor academic performance, and disrupt the classroom (Boysen et al., 2023) and prefer students who are hard-working (Saidah et al., 2019). Teacher-perceived student likability (e.g., the degree to which a student is liked by teachers) has been shown to significantly influence students' social and academic development. Research indicates that lower levels of perceived likability are associated with increased peer rejection, heightened feelings of loneliness, and a decline in academic performance during elementary school (Hendrickx et al., 2017; Mercer and DeRosier, 2008; Sette et al., 2020). Furthermore, teacher-perceived student likability has been shown to be a key internal mechanism through which student behavior influences peer acceptance (Chang et al., 2004, 2007). Previous studies have focused primarily on the impact of teacher preferences on student outcomes, with fewer investigations into how these preferences influence teachers' own behavior. In this study, we aim to examine the impact of teacher-perceived student likability on teacher empathy and, on basis of the motivated account of empathy (Zaki, 2014), explore the underlying mechanisms and ways to eliminate this effect. Understanding these issues is important for discerning how to enhance teacher empathy as a path toward cultivating a supportive and inclusive learning environment. Notably, although intergroup empathy bias has been widely demonstrated (Yaghoubi Jami and Walker, 2022) and people share group membership on basis of their emotional preferences (e.g., people who are liked are identified as ingroup members), highly emotional preferences are not inevitably related to whether the target is inside or outside the group. Therefore, the effects of group membership on empathy are beyond the scope of this paper.

### 1.1 Teacher-perceived student likability and teacher empathy

The perception-action model provides a foundational theoretical framework for understanding the emergence of empathy (Preston, 2007). According to this model, when individuals observe others' emotions or behaviors, their brains automatically activate corresponding neural pathways, as if they were experiencing the same emotions themselves. However, this response is not indiscriminate but rather modulated by relationships between the empathizer and target (e.g., affective De Vignemont and Singer, 2006; Cuff et al., 2016). Some empirical studies have also shown that individuals exhibit higher levels of empathy toward liked others than toward disliked others (Bucchioni et al., 2015; Wang et al., 2014). Specifically, a study revealed that participants displayed stronger feedback-related negativity (FRNs, an ERP component that is generally more pronounced for negative outcomes of our own performance) when observing likable confederates' losses than when observing unlikable confederates' losses (Wang et al., 2014). Another study reported that higher pain ratings were attributed to loved persons than to hated persons when the participants were asked to imagine that someone they love or hate is in a painful situation (Bucchioni et al., 2015).

While the existing research has suggested the impact of perceived likability on empathy in general populations, the teacher-student relationship constitutes a distinctive educational interpersonal dynamic that may exhibit unique patterns of emotional interaction. As members of a helping profession, teachers are professionally obligated to understand, care for, and facilitate the students' development equally (Osguthorpe, 2008). Therefore, it remains to be explored whether perceived likability affects empathy within the teacher-student relationship. Exploring this issue has important implications for fostering more equitable teacher-student interactions and enhancing the emotional climate in the classroom.

### 1.2 The roles of emotional exhaustion and empathic motivation

If teachers demonstrate differential empathy toward liked vs. disliked students, what are the underlying mechanisms driving this disparity, and how can such bias be mitigated? Empathy is not an automatic response to others' suffering; rather, it is highly context-dependent (De Vignemont and Singer, 2006). According to the motivated account of empathy, context indirectly influences empathy by shaping an individual's motives (Zaki, 2014). Empathic motives are goal-directed internal forces that drive individuals either toward or away from empathy (Weisz and Zaki, 2018). These motives are guided by subjective evaluations of the benefits and costs associated with empathizing. Individuals tend to avoid empathy-eliciting situations when empathy comes at a cost (e.g., requires significant cognitive or emotional resources, Cameron et al., 2019). Conversely, individuals are more likely to approach empathy-eliciting situations when empathy bring benefits (e.g., foster meaningful connections with others, Ferguson et al., 2020).

Previous empirical studies have also shown that changing people's motivations to empathize can shape empathic outcomes (Weisz et al., 2021; Weisz and Zaki, 2017). For example, one study indicated that liberals wanted to feel more empathy and experienced more empathy than did conservatives do and that the relationship between political ideology and empathic reactions is mediated by empathic motivation (Hasson et al., 2018). In the specific context of teacher empathy, another study showed that teachers who exhibit a malleable mindset with respect to the ability of students also exhibited greater empathic motivation, thus leading to a stronger experience of empathy (Ge et al., 2021). Additionally, some studies have shown that interventions based on empathic motivation can effectively enhance individuals' empathic responses (Hess et al., 2017; Weisz et al., 2021).

Emotional exhaustion, the depletion of emotional resources and energy (Chang, 2009), is particularly common during empathy. Empathy requires individuals to invest significant emotional resources to understand and share others' feelings while regulating their own emotions to avoid being overwhelmed. This continuous emotional demand often leads to exhaustion (Wróbel, 2013). Emotional exhaustion is frequently observed in high-stakes settings, such as clinical environments. For example, medical practitioners, including physicians, are chronically exposed to the suffering and distress of patients, which can take a significant emotional toll and result in severe exhaustion (Gleichgerrcht and Decety, 2013, 2014). To mitigate this emotional cost, physicians often downregulate their empathic responses, reducing their own distress (Cheng et al., 2007; Decety et al., 2010). Similarly, research has shown that individuals anticipate higher levels of emotional exhaustion when faced with help requests from stigmatized individuals (e.g., drug users), leading them to dehumanize others as a coping strategy to avoid exhaustion (Cameron et al., 2016). These findings collectively highlight how emotional exhaustion serves as a critical driver of empathy avoidance.

Teaching carries high emotional demands, often leading to teacher burnout (Naring et al., 2012; Tuxford and Bradley, 2015). Previous research have shown that teacher-student relationships are closely linked to teachers' emotional exhaustion (Cui, 2022; Wang et al., 2024). When teachers perceive negativity or a lack of rapport in their relationships with their students (e.g., conflictual relationships), they are more likely to experience emotional frustration, fatigue, and strain (Corbin et al., 2019). Likewise, when teachers empathize with students they dislike, they may also experience heightened emotional exhaustion. Since empathy is a prosocial emotion, it may conflict with their true emotions (e.g., annoyance or resentment) of the students. In such situations, teachers may need to cope through surface acting (suppressing and masking their true emotions to appear empathetic) or deep acting (actively adjusting their inner emotions to genuinely experience empathy). However, excessive emotional labor depletes psychological resources, ultimately leading to greater emotional exhaustion (Naring et al., 2012; Wróbel, 2013).

Therefore, we speculate that the possible mechanism for the likable empathy bias includes the fact that teachers anticipate experiencing more emotional exhaustion in empathy situations involving disliked students, which decreases teachers' motivation for empathy and thus leads to a low level of empathy experiences regarding disliked students. If the disparities in teachers' empathic motivation toward disliked and liked students represent a potential mechanism of likable empathy bias, then we believe that interventions targeting empathic motivation may provide a method for mitigating this bias.

### 1.3 The present study

Our research consists of two studies. Study 1 examines whether teacher-perceived student likability affects teacher empathy and whether the decreased empathic motivation resulting from high anticipated emotional exhaustion when encountering empathy situations involving disliked students could account for this effect. Study 2 examines whether the interventions targeting empathic motivation could improve teachers' empathy toward disliked students, thereby mitigating likable empathy bias.

Our research offers the following innovations: First, empathy biases have been extensively studied in general social relationships (Fabi and Leuthold, 2018; Zhao et al., 2021), yet their presence in teacher-student interactions remains largely unexplored. Given that teachers are professionally obligated to care for and treat all students equitably (Osguthorpe, 2008), it is unclear whether their empathy is influenced by emotional preferences. Second, we further investigated the underlying mechanisms of likable empathy bias among teachers based on the motivated account of empathy (Zaki, 2014), specifically, we hypothesize that teachers' anticipation of emotional exhaustion when empathizing with disliked students reduces their empathic motivation, ultimately diminishing empathy. We also propose that interventions targeting empathic motivation could serve as an effective means of mitigating this bias. This is a critical step toward equitable teacher-student interactions.

## 2 Study 1

**Figure 1:**
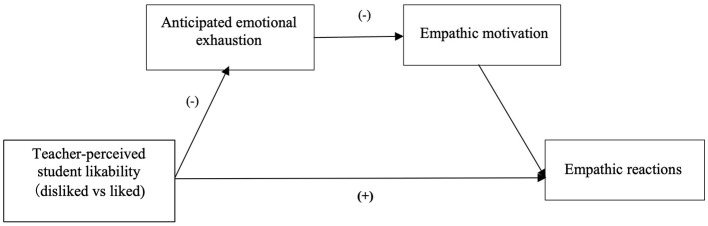
The theoretical model.

The purpose of Study 1 is to examine whether teachers differ in terms of their anticipated emotional exhaustion, empathic motivation and empathic reactions when they encounter empathy situations involving disliked students and liked students. Mediation analyses are also conducted to examine whether the anticipated emotional exhaustion and empathic motivation serially mediate the relationship between teacher- perceived student likability and teacher empathy. Compared with liked students, teachers are expected to anticipate greater emotional exhaustion and lower motivation when encountering empathy situations involving disliked students, and teachers will show lower empathy for disliked students than for liked students. Anticipated emotional exhaustion and empathic motivation will serially mediate the relationship between teacher-perceived student likability and teacher empathy. The theoretical model of the mediation pathway is shown in Figure 1.

### 2.1 Method

#### 2.1.1 Participants and design

A total of 138 primary and middle school teachers from Henan and Hunan Provinces in China participated in the study via an online platform (https://www.wjx.cn). This study employed a single factor between design featuring teacher-perceived student likability (liked, disliked). The sample size was determined on the basis of a power analysis (*f* = 0.25, α = 0.05, 1 – β = 0.8) to require at least 128 participants. The participants were randomly assigned to one of two conditions. Independent sample *t* tests revealed that the two groups did not significantly differ in terms of age (*t* = −1.09, *p* = 0.28), years of teaching experience (*t* = −1.34, *p* = 0.18) or empathy ability (*t* = −1.69, *p* = 0.09). A chi-square test also revealed that the two groups were not significantly different in terms of sex (χ^2^ = 0.37, *p* = 0.54), status as a head teacher (χ^2^ = 0.26, *p* = 0.61) or grade taught (χ^2^ = 0.03, *p* = 0.86). Table 1 provides the detailed demographic information concerning the two groups of participants. This study protocol was approved by the Ethics Committee of Hunan First Normal University in China (ID: 202201001).

**Table 1:** Demographic information for Study 1.

**Demographic variable**		**Group 1 (69)**	**Group 2 (69)**	**Total**
Sex	Female	55	52	107
	Male	14	17	31
Age (years)	Range 20–29	23	12	
	*M (SD)*	26.8 (1.6)	25.3 (2.5)	
	Range 30–39	19	18	
	*M (SD)*	35.6 (2.7)	34.9 (3.2)	
	Range 40–49	22	35	
	*M (SD)*	45.1 (2.9)	43.6 (3.1)	
	Range 50–60	5	4	
	*M (SD)*	52.9 (2.3)	53.0 (2.5)	
Teaching experience (years)	Range	1–37	1–37	
	*M (SD)*	14.9 (11.2)	17.2 (9.1)	
Head teacher	Yes	34	37	71
	No	35	32	67
Grades taught	Grades 1–6	38	37	75
	Grades 7–9	31	32	63

#### 2.1.2 Teacher-perceived student likability manipulation

To manipulate teacher-perceived student likability, we systematically constructed two student descriptions on the basis of commonly favored and disfavored personality traits identified through a preliminary survey. In this survey, 383 primary and middle school teachers were asked to list three personality traits that they most and least preferred in students. The three most frequently mentioned favored traits were *diligent, honest*, and *kind*, while the three most frequently mentioned disfavored traits were *lazy, lying*, and *selfish*. Using these findings, we developed two vignettes. In the liked condition, the student was described as diligent, honest, and kind. In the disliked condition, the student was characterized as lazy, lying, and selfish. This approach ensured that our manipulation was grounded in teachers' commonly shared preferences rather than arbitrary selections. Furthermore, by systematically varying only the described personality traits while keeping other contextual factors constant (e.g., sex and grade), we aimed to isolate the effect of teacher-perceived student likability on empathy.

#### 2.1.3 Procedures

The participants were told that the purpose of the study was to examine their reactions to negative events pertaining to students. The participants were informed that they were about to read a text describing an event in which a student experienced betrayal from a close friend, resulting in profound sadness. They were asked to indicate their attitude toward this event. Prior to reading the text, the participants read a vignette introducing the student. Next, the participants were asked to answer some questions to indicate their anticipated emotional exhaustion and motivation to exhibit empathy toward the student. They were then presented with the text and reported their empathic reactions toward the student. To check whether the teacher-perceived student likability manipulation was successful, they were then asked to rate how much they liked the student. Finally, the participants provided their demographic information and completed the Empathy Scale for Teachers (EST) to measure their empathy ability (Wang et al., 2022).

#### 2.1.4 Measures

##### 2.1.4.1 Teacher-perceived student likability manipulation check

One item was used to examine the effectiveness of the teacher-perceived student likability manipulation: “*To what extent do you like this student?*” (from 1 = greatly dislike to 7 = greatly like). The measure was adapted from previous studies on teachers' liking of students and demonstrated good test-retest reliability (Chang et al., 2004, 2007).

##### 2.1.4.2 Anticipated emotional exhaustion

Three items adapted from the research conducted by Cameron et al. (2016) (i.e., “*How emotionally exhausting and draining will it be to learn about a negative event involving this student?*”; “*How emotionally exhausting and draining will it be to experience the negative emotion of this student?*”; and “*How emotionally exhausting and draining will it be to empathize with this student?*”) were used to measure anticipated emotional exhaustion. The participants responded on a 7-point scale (1 = not at all, 7 = to a large extent). The internal consistency reliability analysis revealed that the Cronbach's α was 0.89.

##### 2.1.4.3 Empathic motivation

Empathic motivation reflects an individual's willingness to empathize with others. Three items adapted from the research of Ge et al. (2021) (i.e., “*To what extent do you want to put yourself in this student's shoes to understand his feelings*?”, “*To what extent do you want to share the feelings of this student*?”, and “*To what extent do you want to empathize with this student?*”) were used to measure empathic motivation before reading the text. The participants responded on a 7-point scale (1 = not at all, 7 = to a large extent). The internal consistency reliability analysis revealed that the Cronbach's α was 0.83.

##### 2.1.4.4 Empathic reaction

Three items adapted from the previous studies (Fan and Han, 2008; Hasson et al., 2018) (i.e., “*How sad do you think this student was when this event happened*?”, “*To what extent could you experience this student's feelings?*” and “*To what extent do you feel sad for him experiencing such a situation?*”) were used to measure empathic reactions immediately after the text was read. The participants responded on a 7-point scale (1 = not at all, 7 = to a large extent). The internal consistency reliability analysis revealed that the Cronbach's α was 0.82.

##### 2.1.4.5 Empathy ability

The EST is a tool for measuring the empathy ability of teachers in relation to their pupils, and it includes 19 items. The internal consistency reliability analysis revealed that the Cronbach's α was 0.82. The EST was used to measure the participants' empathy ability in this study.

#### 2.1.5 Statistical analysis

The statistical analyses were conducted using SPSS 27.0 and Mplus 8.3. In SPSS, the following procedures were performed: (1) assess measurement reliability using Cronbach's α coefficients; (2) test the effects of the independent variable on the mediators and dependent variable through one-way ANCOVAs; and (3) diagnosing multicollinearity among the predictors (e.g., anticipated emotional exhaustion and empathic motivation) by calculating variance inflation factors (VIFs) with a conservative threshold of VIF <3 (O'Brien, 2007). In Mplus 8.3, confirmatory factor analyses (CFAs) were conducted to evaluate the model fit and the discriminant validity of the variables, with fit indices (χ^2^/*df* , CFI, TLI, RMSEA, SRMR) benchmarked against established thresholds (Fonseca-Pedrero, 2012). Finally, serial mediation hypotheses were tested using Hayes' (2013) PROCESS macro (Model 6) in SPSS, with parameters estimated via 5,000 bias-corrected bootstrap resamples. Effects were considered statistically significant if the 95% confidence intervals excluded zero.

### 2.2 Results

#### 2.2.1 CFAs and multicollinearity test

We tested several factor measurement models and summarize the results in Table 2. As shown in Table 2, the fit index of the initial model (M0: three-factor model) met the model fit criteria (χ^2^/*df* < 5, CFI > 0.90, TLI > 0.90, RMSEA <0.08, SRME <0.08); and was superior to those of alternative models. This finding indicates that the initial model has good construct validity and that the variables exhibit satisfactory discriminant validity. Multicollinearity diagnostics indicated that the VIFs for both predictor variables—anticipated emotional exhaustion and empathic motivation—were 1.31, significantly below the conservative threshold of 3.0. This confirms the absence of multicollinearity-related bias in the regression estimates.

**Table 2:** Comparisons of measurement models.

**Models**	**χ^2^**	***df***	**χ^2^/*df***	**CFI**	**TLI**	**RMSEA**	**SRME**
M0: three factors	41.11	24	1.71	0.96	0.98	0.07	0.04
M1: two factors	147.22	26	5.66	0.76	0.83	0.18	0.11
M2: two factors	236.47	26	9.10	0.58	0.70	0.24	0.20
M3: two factors	290.13	26	11.16	0.47	0.62	0.27	0.23
M4: one factors	273.52	27	10.13	0.33	0.50	0.31	0.18

M0: three factors (anticipated emotional exhaustion, empathic motivation, empathic reactions); M1: two factors (empathic motivation and empathic reactions combined to one factor); M2: two factors (anticipated emotional exhaustion and empathic motivation combined to one factor); M3: two factors (anticipated emotional exhaustion and empathic reactions combined to one factor); M4: one factor (all variables combined to one factor).

#### 2.2.2 Teacher-perceived student likability manipulation check

To test the effectiveness of the teacher-perceived student likability manipulation, we conducted a one-way ANOVA with teacher-perceived student likability as the independent variable and teachers' liking ratings for the student as the dependent variable. The results showed that the participants rated the student in the disliked condition (*M* = 2.62, *SD* = 1.19) as less likable than they did the student in the liked condition [*M* = 6.39, *SD* = 0.97, *F*_(1, 136)_ = 414.72, *p* < 0.001, $\eta_{p}^{2}$ = 0.75].

#### 2.2.3 Main effects

##### 2.2.3.1 Anticipated emotional exhaustion

To examine whether teacher-perceived student likability influences anticipated emotional exhaustion prior to encountering with empathy situations, we conducted a one-way ANCOVA featuring teacher-perceived student likability (liked, disliked) as a between-subjects factor. The sex (1 = male, 2 = female), age, years of teaching experience, whether the respondent was a head teacher (1 = yes, 2 = no), grade taught (1 = 1–6 grade, 2 = 7–9 grade) and empathy ability were covariates. The results showed that participants anticipated more emotional exhaustion from experiencing empathy for disliked students (*M* = 3.99, *SD* = 1.29) than they did for liked students [*M* = 3.07, *SD* = 1.65, *F*_(1, 130)_ = 15.78, *p* < 0.001, $\eta_{p}^{2}$ = 0.11].

##### 2.2.3.2 Empathic motivation

We repeated the analyses discussed above to predict empathic motivation toward the students. The participants were less motivated to empathize with disliked students (*M* = 4.66, *SD* = 1.25) than they were to empathize with liked students [*M* = 6.12, *SD* = 0.71, *F*_(1, 130)_ = 70.41, *p* < 0.001, $\eta_{p}^{2}$ = 0.35].

##### 2.2.3.3 Empathic reaction

We ran similar analyses to predict subsequent empathic reactions toward students upon reading about the a negative event. The participants experienced less empathy for disliked students (*M* = 5.30, *SD* = 1.36) than for liked students [*M* = 5.92, *SD* = 0.91, *F*_(1, 130)_ = 9.24, *p* = 0.003, $\eta_{p}^{2}$ = 0.07].

#### 2.2.4 Mediation analysis

We tested whether the link between teacher-perceived student likability and empathic reactions was mediated by anticipated emotional exhaustion and empathic motivation. Accordingly, we conducted a serial mediation analysis employing the PROCESS bootstrapping macro (Model 6; 5,000 iterations). The model was designed to include teacher-perceived student likability as the independent variable, anticipated emotional exhaustion as the first mediator, empathic motivation as the second mediator, and empathic reactions as the dependent variable. Demographic variables and the EST score were included in the model as control variables.

**Figure 2:**
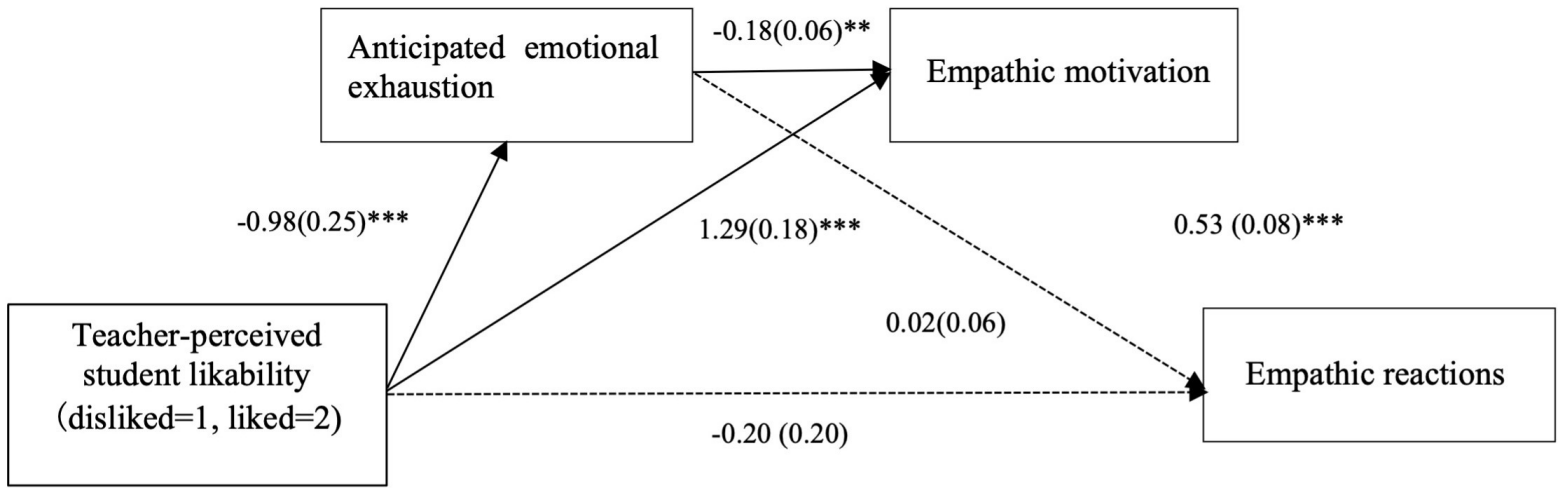
Mediation model of teacher-perceived student likability, anticipated emotional exhaustion, empathic motivation and empathic reactions. ***p < 0.001.

The results revealed that the total effect of teacher-perceived student likability on empathic reactions was significant (*B* = 0.56, 95% CI = [0.20, 0.93], *t* = 3.04, *p* = 0.003). However, the direct effect of teacher-perceived student likability on empathic reactions was non-significant (*B* = −0.20, 95% CI = [−0.60, 0.21], *t* = −0.96, *p* = 0.34) when anticipated emotional exhaustion and empathic motivation were included as serial mediators. The indirect effect through anticipated emotional exhaustion was non-significant (*B* = −0.02, 95% CI = [−0.14, 0.10]). The indirect effect through empathic motivation was significant (*B* = 0.68, 95% CI = [0.37, 1.02]). The indirect effect through both of two mediators was significant (*B* = 0.09, 95% CI = [0.03, 0.17]). The path coefficients are shown in Figure 2.

## 3 Study 2

Study 1 revealed that teachers do not empathize equally with liked and disliked students, a disparity that may negatively impact educational outcomes and classroom dynamics. This unequal distribution of empathy underscores the need for targeted interventions to increase teacher emotional engagement with students they dislike. Study 1 revealed two critical pathways through which low perceived likability impaired teachers' empathy: a significant standalone mediation via empathic motivation (indirect effect = 0.68) and a smaller yet significant chained mediation through anticipated emotional exhaustion and empathic motivation (indirect effect = 0.09). This pattern highlights empathic motivation as the dominant and proximal mechanism driving empathy deficits. These findings underscore the potential value of interventions aimed at enhancing empathic motivation as a means to mitigate empathy bias. Consequently, Study 2 is designed to test an intervention targeting empathic motivation to determine whether it can effectively mitigate the decline in empathy toward disliked students.

According to the expectancy-value model of emotion regulation, people are motivated to emote in ways they expect to be useful to them, regardless of immediate contextual demands (Tamir et al., 2015). For example, people may be motivated to become angry when they believe that anger is useful, even when there is nothing to be angry about. According to this theory, even in aversive situations (e.g., interacting with disliked students), a teacher would be motivated to empathize as long as she thinks that empathy is beneficial in that context. As such, we manipulated the perceived utility of empathy by advising teachers that empathy could help them develop effective strategies for addressing students' issues. We hypothesize that manipulating the perceived utility of empathy can effectively activate teachers' empathic motivation; increased motivation would mediate improvements in empathic reactions; and the empathy difference between liked and disliked students would be significantly lower in the intervention condition than in the control condition. By linking the findings of Study 1 with the manipulation in Study 2, we aimed to translate the identified psychological mechanism into a practical strategy to enhance teacher empathy.

### 3.1 Method

#### 3.1.1 Participants and design

A total of 221 primary and middle school teachers from Henan and Hunan provinces in China participated in the study via an online platform (https://www.wjx.cn). This study employed a 2 (teacher-perceived student likability: liked, disliked) × 2 (experimental condition: intervention condition, control condition) between-subjects design. The sample size was determined on the basis of a power analysis (*f* = 0.25, α = 0.05, 1 – β = 0.8) to require at least 179 participants. The participants were randomly assigned to one of four conditions. One-way ANOVA revealed no significant differences among the four groups in terms of age (*F* = 1.64, *p* = 0.18), years of teaching experience (*F* = 1.83, *p* = 0.14) or empathy ability (*F* = 1.15, *p* = 0.33). A chi-square test revealed that the four groups exhibited no significant differences in terms of sex (χ^2^ = 1.95, *p* = 0.58) or grade taught (χ^2^ = 1.88, *p* = 0.60) and only marginally significant differences in terms of whether they were head teachers (χ^2^ = 7.58, *p* = 0.06). Table 3 provides detailed demographic information concerning the four groups of participants. This study protocol was approved by the Ethics Committee of Hunan First Normal University in China (ID: 202201001).

**Table 3:** Demographic information for study 2.

**Demographic variable**		**Group1 (62)**	**Group2 (61)**	**Group3 (51)**	**Group4 (50)**	**Total**
Sex	Female	15	13	11	7	46
	Male	47	45	40	43	175
Age (years)	Range 20–29	28	33	16	21	98
	*M (SD)*	25.6 (2.4)	24.6 (2.2)	26.6 (2.1)	24.7 (2.2)	
	Range 30–39	14	9	10	11	44
	*M (SD)*	35.9 (2.3)	33.3 (1.7)	35.7 (3.2)	34.6 (3.0)	
	Range 40–49	13	10	21	10	54
	*M(SD)*	44.4(2.6)	44.5(2.5)	42.8(2.7)	44.2(2.5)	
	Range 50–60	7	6	4	8	25
	*M (SD)*	52.0 (3.0)	53.0 (3.0)	52.5 (2.9)	52.9 (2.8)	
Teaching experience (years)	Range	0.5–38	0.5–39	0.5–34	0.5–40	
	*M (SD)*	12.8 (11.4)	10.1 (11.5)	14.9 (9.6)	13.4 (12.0)	
Head teacher	Yes	30	18	25	15	88
	No	32	40	26	35	133
Grades taught	Grades 1–6	24	28	19	19	90
	Grades 7–9	38	30	32	31	131

#### 3.1.2 Procedures

This study employed a randomized experimental design to implement an intervention by activating teachers' empathic motivation though manipulating the perceived utility of empathy. The participants were randomly assigned to the “intervention condition” or “control condition” to examine the moderating effect of motivational activation on empathy likable bias among teachers. The procedure was as follows:

The participants were told that the purpose of the study was to gather effective solutions to address students' problems. They were informed that they would read a text describing an event in which a student experienced betrayal from a close friend, resulting in profound sadness. They were asked to provide potential solutions to cope with this problem and be rewarded for good performance.

We primed the motivation for empathy using a previously used and validated procedure (Porat et al., 2016; Tamir et al., 2015). In the intervention condition, participants were informed that before reading the text, they would have access to tips provided by previous participants who had performed well in the study. Three fictional tips were presented to the participants. One tip was unrelated to empathy (i.e., “*What helped me the most was reading the text carefully and keeping my mind focused”*), but two of the tips suggested that empathy may be useful for proposing solutions (i.e., “*To propose the best solution, it is better to adopt the perspective of the student and imagine how the student's suffering affects his life*”; “*While reading, I tried to understand the thinking of the student and feel what he feels, which helped me a lot*”). To ensure that participants processed the tips without feeling compelled to adhere to them, they were informed that we needed their assistance in evaluating the tips for relevance. As such, participants were asked to select two out of the three tips they found sensible and explain their choices in their own words. The participants in the control condition did not have access to any tips.

Next, the participants read a vignette intended to manipulate perceived student likability, as in Study 1. The participants then read the same text as employed in Study 1 and rated their empathic motivation and empathic reaction to the student. Finally, the participants provided their demographic information and completed the EST.

#### 3.1.3 Measures

The measures of the teacher-perceived student likability manipulation check, empathic reaction (α = 0.80) and empathy ability (α = 0.84) were the same as those used in Study 1. The measure of the empathic motivation manipulation check (α = 0.92) was the same as the measure of empathic motivation used in Study 1.

#### 3.1.4 Statistical analysis

The statistical analyses were conducted using SPSS 27.0, and the following procedures were performed: (1) a one-way ANOVA was performed to test the effectiveness of the perceived student likability manipulation and the empathic motivation priming; (2) Hayes' (2013) PROCESS macro (Model 4), with parameters estimated via 5,000 bias-corrected bootstrap resamples, was used to test whether the increase in empathic motivation mediated the relationship between the experimental condition and enhanced empathic responses. Effects were considered statistically significant if the 95% confidence intervals excluded zero. (3) A two-way ANCOVA was conducted to analyze the main effects of student likability and experimental condition, as well as their interaction effects. Simple effects analyses were performed to further explore significant interactions.

### 3.2 Results

#### 3.2.1 Teacher-perceived student likability manipulation check

The results showed that the participants rated the student in the disliked condition (*M* = 2.53, *SD* = 0.80) as less likable than they did the student in the liked condition [*M* = 5.89, *SD* = 0.88, *F*_(1, 219)_ = 881.19, *p* < 0.001, $\eta_{p}^{2}$ = 0.80].

#### 3.2.2 Empathic motivation manipulation check

The results revealed that the participants in the intervention condition (*M* = 5.93, *SD* = 0.99) were significantly more motivatied to experience empathy for the students than the participants in the control condition were [*M* = 5.23, *SD* = 1.37, *F*_(1, 219)_ = 18.45, *p* < 0.001, $\eta_{p}^{2}$ = 0.08].

#### 3.2.3 Mediation analysis

The results revealed that the total effect of the experimental condition on empathic reactions was non-significant (95% CI = [−0.06, 0.52]). The direct effect of the experimental condition on empathic reactions was non-significant (95% CI = [−0.38, 0.13]) when empathic motivation was included as a mediator. The indirect effect through empathic motivation was significant (*B* = 0.35, 95% CI = [0.18, 0.56]).

#### 3.2.4 Empathic reactions

**Figure 3:**
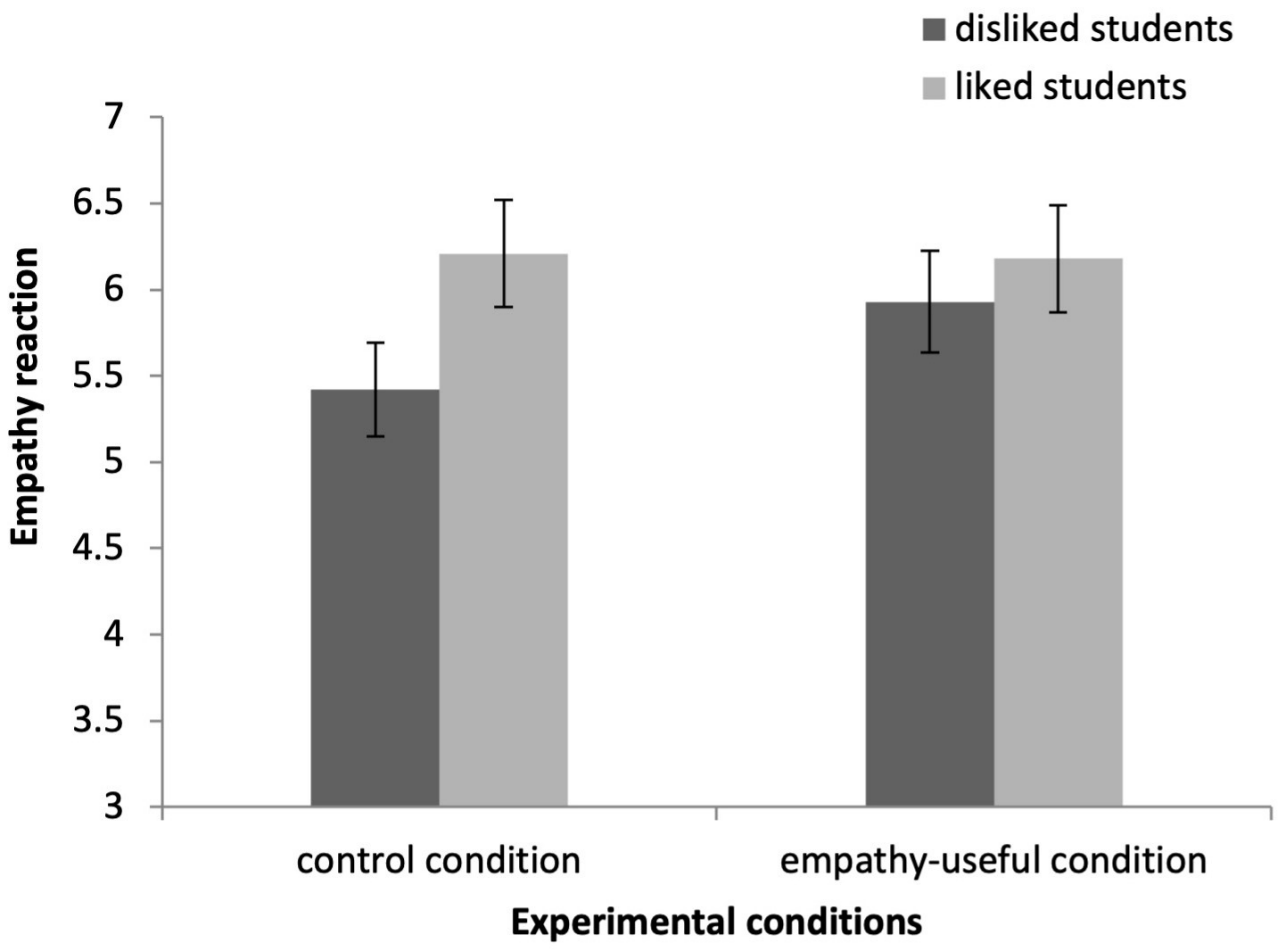
Empathic reaction as a function of the teacher-perceived student likability and empathic motivation. Error bars represent ±1 standard error.

We conducted a two-way ANCOVA with teacher-perceived student likability and experimental condition as between-subjects factors and included demographic variables and empathy ability as covariates. The main effect of teacher-perceived student likability was significant [*F*_(1, 211)_ = 9.64, *p* = 0.002, $\eta_{p}^{2}$ = 0.04]. The main effect of the experimental condition was not significant [*F*_(1, 211)_ = 2.07, *p* > 0.05]. The interaction effect between teacher-perceived student likability and the experimental condition was marginally significant [*F*_(1, 211)_ = 3.37, *p* = 0.07, $\eta_{p}^{2}$ = 0.02]. The participants in the control condition experienced less empathy for the disliked students (*M* = 5.42, *SD* = 1.47) than they did for the liked students (*M* = 6.21, *SD* = 0.79, *p* < 0.001). The participants in the intervention condition did not experience empathy differently for the disliked students (*M* = 5.93, *SD* = 1.02) and the liked students (*M* = 6.18, *SD* = 0.79, *p* = 0.27). For the disliked students, the participants experienced more empathy in the intervention condition than they did in the control condition (*p* < 0.05). For the liked students, the participants exhibited no differences in terms of their experience of empathy between the intervention and control conditions (*p* > 0.05). The results are shown in Figure 3.

## 4 General discussion

Across these two studies, this research examines whether teacher-perceived student likability affects teacher empathy as well as the mechanism underlying this effect (study 1) and ways of mitigating this empathy bias (study 2). Study 1 found that teachers exhibited less empathy for disliked students than they do for liked students and that anticipated emotional exhaustion and empathic motivation serially mediate the relationship between teacher-perceived student likability and teacher empathy. Study 2 revealed that the interventions targeting empathic motivation enhanced teachers' empathy toward their disliked students; therefore, the effect of perceived student likability on teacher empathy is eliminated. These findings suggest that empathic motivation plays a crucial role in likable empathy bias among teachers in that it not only functions as a key mechanism underlying this bias but also emerges as a potential pathway for mitigating such bias.

### 4.1 The likable empathy bias among teachers

Our study revealed a significant likable empathy bias in teacher-student interactions, which is consistent with findings in the general population (Bucchioni et al., 2015; Wang et al., 2014). This suggests that even within educational contexts governed by explicit professional norms, teachers remain influenced by subjective affective preferences. Our study also addresses, to some extent, the methodological limitations of previous research. Wang et al. (2014) manipulated perceived likability using a social exchange game where confederates played fairly (likable) or unfairly (unlikable), potentially confounding perceived likability with retaliatory motivations (Singer et al., 2006). Bucchioni et al. (2015) asked participants to nominate loved or hated individuals, but the groups differed significantly in interpersonal distance, as “loved” individuals were often family members or partners, while “hated” individuals were typically bosses or teachers. Our study manipulates teachers' perceptions of student likability using personality trait descriptions derived from a survey that identified the traits teachers most and least appreciate in students within real educational settings. This approach not only enhances ecological validity but also minimizes potential confounding factors such as interpersonal distance and retaliatory motives.

### 4.2 The underlying mechanism of the likable empathy bias among teachers

Our study further elucidates the underlying mechanism of the likable empathy bias among teachers, showing that teachers experience higher levels of anticipated emotional exhaustion when empathizing with disliked students, which in turn reduces their empathic motivation and ultimately lowers their empathy levels. This finding aligns with the motivated account of empathy, which posits that empathy is not solely a matter of ability (Baron-Cohen and Wheelwright, 2004) but also shaped by one's willingness to engage with others' emotions, driven by perceived costs and benefits (Keysers and Gazzola, 2014; Zaki, 2014). The role of empathic motivation in empathy has been well established in numerous studies (Cameron et al., 2019; Cameron and Payne, 2011; Ferguson et al., 2020; Weisz and Zaki, 2018). For example, studies have shown that liberals tend to exhibit higher levels of empathy than conservatives do and that teachers with a malleable mindset regarding students' abilities show greater empathy than do those with a fixed mindset. In both cases, empathic motivation plays a crucial mediating role. However, these studies have not explored which factors or processes are responsible for differences in empathic motivation. Consistent with previous research, our study also confirms the role of empathic motivation in the experience of empathy. Furthermore, we identified the underlying processes that drive differences in teachers' empathic motivation toward liked and disliked students.

Emotional exhaustion is known to deplete affective resources, making it difficult for individuals to sustain emotional engagement in demanding situations. As a self-protective mechanism, individuals may withdraw from emotionally taxing interactions to conserve their remaining psychological energy (Cameron et al., 2016; Cheng et al., 2007; Decety et al., 2010). In educational settings, emotional exhaustion is a central component of teacher burnout, significantly influencing their job satisfaction, turnover intentions, and mental wellbeing (Jeon et al., 2018; Lee, 2019; Rajendran et al., 2020). To alleviate emotional strain and prevent burnout, teachers may unconsciously limit their empathy toward disliked students, even when professional norms and ethical guidelines emphasize the importance of treating all students impartially. This may because empathizing with disliked students often requires greater emotional labor, further depleting teachers' emotional resources (Saloviita and Pakarinen, 2021; Skaalvik and Skaalvik, 2017). This pattern of selective empathy suggests that the likable empathy bias among teachers is not merely a reflection of personal favoritism but rather a regulatory strategy to manage the emotional burdens of their profession. By prioritizing their own emotional wellbeing, teachers may unknowingly engage in differential treatment of students, potentially reinforcing existing disparities in teacher-student relationships and student outcomes.

### 4.3 The intervention for mitigating likable empathy bias among teachers

In teaching practice, students who are less favored by teachers often exhibit lower academic performance and face more behavioral challenges (Boysen et al., 2023; Saidah et al., 2019). These students typically require additional care and support from their teachers (Okonofua et al., 2016; Waajid et al., 2013). Low levels of empathy and unfair treatment may exacerbate these students' issues, creating a vicious cycle. Therefore, it is crucial to implement effective measures to increase teachers' empathy toward disliked students. The findings from Study 1 suggest that interventions targeting empathic motivation could be an effective strategy to mitigate teachers' likable empathy bias. Building on this, Study 2 examined the impact of an empathic motivation intervention by manipulating the perceived utility of empathy. The results of Study 2 revealed that enhancing the perceived utility of empathy successfully activated teachers' empathic motivation. Furthermore, as expected, teachers' empathy toward disliked students increased, and the effect of perceived student likability on teacher empathy was eliminated when empathic motivation was primed. Traditionally, most empathy interventions, such as perspective-taking exercises (Ames et al., 2008) and mentalizing training (Riess et al., 2012), have focused on enhancing “ability”, The motivated account of empathy suggests a complementary approach—boosting individuals' motivation to engage empathically. The effectiveness of empathy interventions based on motivation has been supported by previous research (Weisz et al., 2021; Weisz and Zaki, 2017). Our study also confirms that enhancing empathic motivation is an effective way to improve teachers' empathy toward disliked students. It provides a practical and scalable strategy for mitigating likability empathy bias in teacher-student interactions, which is essential for promoting equitable and fair teaching practices.

### 4.4 Practical implications

This research has important implications for educational practice. First, teachers should be aware that their personal emotions may influence their interactions with students. To prevent the emergence of likable empathy bias from the outset, teachers should consciously manage their emotion through self-reflection and emotion regulation, ensuring that every student receives the attention and support they deserve in a fair environment. Second, schools should provide support systems to help teachers manage emotional fatigue. Teachers may experience emotional exhaustion when dealing with disliked students, which not only weakens their empathic responses but also affects their overall teaching effectiveness. Therefore, schools can implement measures such as peer support, emotional counseling, and stress management to help teachers reduce emotional burdens and enhance their empathic abilities. Finally, our research highlights the crucial role of empathic motivation in reducing likable empathy bias. To promote fairness and empathy in the classroom, schools should include empathic motivation interventions in teachers' professional development programs.

### 4.5 Limitations and future directions

The present work is not without limitations. First, the results of Study 2 showed that the interaction between the experimental condition and perceived student likability only reached marginal significance, possibly because of the intervention processes we used. Our interventions were designed to strengthen empathic approach motives, but the likable empathy bias in teachers is likely to stem from their routine avoidance of empathizing with disliked students. Additionally, Study 2 employed a one-time intervention using a randomized controlled experiment rather than a long-term intervention, which may have limited the effect. Future research could focus on extended multisession interventions that subtly reduce avoidance motives to better understand the effects of the motivational intervention. Second, we used a hypothetical scenario to examine the likable empathy bias in teachers and the effects of empathic motivation interventions. Therefore, the generalizability of the findings to real educational settings should be approached with caution. Future research should employ real-world classroom-based studies to validate these findings and could also further explore how other factors, such as teacher burnout and stress levels, moderate this bias in real classroom contexts. For instance, teachers with lower levels of emotional exhaustion may be less prone to experiencing this bias, which could make them more empathetic regardless of perceived student likability. Third, all the measures used in this research were based on self-reports. The participants may have responded in a socially desirable manner. Future studies should combine behavioral indicators (ie, empathic accuracy) or physiological indexes (ERP component) to test whether these results still hold. Fourth, in Study 2, the lack of a time interval between the intervention and empathy measurement may allow for placebo effects. Although the effectiveness of the emotional-usefulness manipulation procedure used in Study 2 has been validated in previous research (Porat et al., 2016; Tamir et al., 2015), future studies could minimize the influence of placebo effects by incorporating a delayed measurement or a placebo control group (e.g., receiving tips unrelated to empathy). Fifth, our study only measured overall empathy without examining its sub-components. Future research could further explore how perceived student likability affects teachers' cognitive and emotional empathy (Meyers et al., 2019), as well as the impact of empathic motivation interventions on these components. This would provide more targeted recommendations for the intervention and development of teacher empathy.

## 5 Conclusion

Teacher empathy is crucial for both student development and teacher growth. Our findings reveal that teachers exhibit lower empathy toward disliked students compared to liked students. Based on the motivated account of empathy, we identified a sequential mediation effect of anticipated emotional exhaustion and empathic motivation in the relationship between perceived student likability and teacher empathy. Furthermore, our study demonstrates that an intervention based on empathy motivation can effectively improve teachers' empathy for disliked student, thereby mitigating this bias. These findings offer preliminary contributions to fostering teacher empathy and promoting equitable education.

## Data Availability

The original contributions presented in the study are included in the article/supplementary material, further inquiries can be directed to the corresponding author.

## References

[B1] Aldrup K. Carstensen B. Klusmann U. (2022). Is empathy the key to effective teaching? A systematic review of its association with teacher-student interactions and student outcomes. Educ. Psychol. Rev. 34, 1177–1216. 10.1007/s10648-021-09649-y

[B2] Ames D. L. Jenkins A. C. Banaji M. R. Mitchell J. P. (2008). Taking another person's perspective increases self-referential neural processing. Psychol. Sci. 19, 642–644. 10.1111/j.1467-9280.2008.02135.x 18727776

[B3] Baron-Cohen S. Wheelwright S. (2004). The empathy quotient: an investigation of adults with asperger syndrome or high functioning autism, and normal sex differences. J. Autism Dev. Disord. 34, 163–175. 10.1023/B:JADD.0000022607.19833.00 15162935

[B4] Boysen G. A. Isaacs R. A. Chicosky R. L. Delmore E. E. (2023). Intense dislike of students: frequency, causes, effects, and management among college teachers. Scholarsh. Teach. Learn. Psychol. 9:133. 10.1037/stl0000200

[B5] Bucchioni G. Lelard T. Ahmaidi S. Godefroy O. Krystkowiak P. Mouras H.. (2015). Do we feel the same empathy for loved and hated peers? PLoS ONE 10:e0125871. 10.1371/journal.pone.0125871 26024234 PMC4449017

[B6] Cai Y. Yang Y. Ge Q. Weng H. (2023). The interplay between teacher empathy, students' sense of school belonging, and learning achievement. Eu. J. Psychol. Educ. 38, 1167–1183. 10.1007/s10212-022-00637-6

[B7] Cameron C. D. Harris L. T. Payne B. K. (2016). The emotional cost of humanity: anticipated exhaustion motivates dehumanization of stigmatized targets. Soc. Psychol. Pers. Sci. 7, 105–112. 10.1177/1948550615604453

[B8] Cameron C. D. Hutcherson C. A. Ferguson A. M. Scheffer J. A. Hadjiandreou E. Inzlicht M.. (2019). Empathy is hard work: people choose to avoid empathy because of its cognitive costs. J. Exp. Psychol. Gen. 148, 962–976. 10.1037/xge0000595 30998038

[B9] Cameron C. D. Payne B. K. (2011). Escaping affect: how motivated emotion regulation creates insensitivity to mass suffering. J. Pers. Soc. Psychol. 100, 1–15. 10.1037/a0021643 21219076

[B10] Chang L. Liu H. Fung K. Y. Wang Y. Wen Z. Li H.. (2007). The Mediating and moderating effects of teacher preference on the relations between students' social behaviors and peer acceptance. Merrill Palmer Q. 53, 603–630. 10.1353/mpq.2008.0006

[B11] Chang L. Liu H. Wen Z. Fung K. Y. Wang Y. Xu Y.. (2004). Mediating teacher liking and moderating authoritative teachering on chinese adolescents' perceptions of antisocial and prosocial behaviors. J. Educ. Psychol. 96, 369–380. 10.1037/0022-0663.96.2.369

[B12] Chang M-. L. (2009). An appraisal perspective of teacher burnout: examining the emotional work of teachers. Educ. Psychol. Rev. 21, 193–218. 10.1007/s10648-009-9106-y

[B13] Cheng Y. Lin C. P. Liu H. L. Hsu Y. Y. Lim K. E. Hung D.. (2007). Expertise modulates the perception of pain in others. Curr. Biol. 17, 1708–1713. 10.1016/j.cub.2007.09.020 17900903

[B14] Corbin C. M. Alamos P. Lowenstein A. E. Downer J. T. Brown J. L. (2019). The role of teacher-student relationships in predicting teachers' personal accomplishment and emotional exhaustion. J. Sch. Psychol. 77, 1–12. 10.1016/j.jsp.2019.10.001 31837719

[B15] Cuff B. M. P. Brown S. J. Taylor L. Howat D. J. (2016). Empathy: a review of the concept. Emot. Rev. 8, 144–153. 10.1177/1754073914558466

[B16] Cui L. (2022). the role of teacher-student relationships in predicting teachers' occupational wellbeing, emotional exhaustion, and enthusiasm. Front. Psychol. 13:736656. 10.3389/fpsyg.2022.896813 35664194 PMC9162152

[B17] De Vignemont F. Singer T. (2006). The empathic brain: how, when and why? Trends Cognit. Sci. 10, 435–441. 10.1016/j.tics.2006.08.008 16949331

[B18] Decety J. Yang C. Y. Cheng Y. (2010). Physicians down-regulate their pain empathy response: an event-related brain potential study. NeuroImage 50, 1676–1682. 10.1016/j.neuroimage.2010.01.025 20080194

[B19] Fabi S. Leuthold H. (2018). Racial bias in empathy: do we process dark- and fair-colored hands in pain differently? An EEG study. Neuropsychologia 114, 143–157. 10.1016/j.neuropsychologia.2018.04.024 29702161

[B20] Fan Y. Han S. (2008). Temporal dynamic of neural mechanisms involved in empathy for pain: an event-related brain potential study. Neuropsychologia 46, 160–173. 10.1016/j.neuropsychologia.2007.07.023 17825852

[B21] Ferguson A. M. Cameron C. D. Inzlicht M. (2020). Motivational effects on empathic choices. J. Exp. Soc. Psychol. 90:104010. 10.1016/j.jesp.2020.104010

[B22] Fonseca-Pedrero E. (2012). Structural equation modeling with Mplus: basic concepts, applications, and programming. Psicothema 24, 343–344.

[B23] Fu W. Wang C. Chai H. Xue R. (2022). Examining the relationship of empathy, social support, and prosocial behavior of adolescents in China: a structural equation modeling approach. Hum. Soc. Sci. Commun. 9:269. 10.1057/s41599-022-01296-0

[B24] Ge Y. Li W. Chen F. Kayani S. Qin G. (2021). The theories of the development of students: a factor to shape teacher empathy from the perspective of motivation. Front. Psychol. 12:736656. 10.3389/fpsyg.2021.736656 34867618 PMC8635053

[B25] Gleichgerrcht E. Decety J. (2013). Empathy in clinical practice: how individual dispositions, gender, and experience moderate empathic concern, burnout, and emotional distress in physicians. PLoS ONE 8:e61526. 10.1371/journal.pone.0061526 23620760 PMC3631218

[B26] Gleichgerrcht E. Decety J. (2014). The relationship between different facets of empathy, pain perception and compassion fatigue among physicians. Front. Behav. Neurosci. 8:243. 10.3389/fnbeh.2014.00243 25071495 PMC4093939

[B27] Hasson Y. Tamir M. Brahms K. S. Cohrs J. C. Halperin E. (2018). Are liberals and conservatives equally motivated to feel empathy toward others? Pers. Soc. Psychol. Bull. 44, 1449–1459. 10.1177/0146167218769867 29739293

[B28] Hayes A. F. (2013). Introduction to Mediation, Moderation, and Conditional Process Analysis: A Regression-Based Approach. New York, NY: The Guilford Press.

[B29] Hendrickx M. M. H. G. Mainhard T. Boor-Klip H. J. Brekelmans M. (2017). Teacher liking as an affective filter for the association between student behavior and peer status. Contemp. Educ. Psychol. 49, 250–262. 10.1016/j.cedpsych.2017.03.004

[B30] Hess U. Blaison C. Dandeneau S. (2017). The impact of rewards on empathic accuracy and emotional mimicry. Motiv. Emot. 41, 107–112. 10.1007/s11031-016-9590-6

[B31] Jeon L. Buettner C. K. Grant A. A. (2018). Early childhood teachers' psychological wellbeing: exploring potential predictors of depression, stress, and emotional exhaustion. Early Educ. Dev. 29, 53–69. 10.1080/10409289.2017.1341806

[B32] Keysers C. Gazzola V. (2014). Dissociating the ability and propensity for empathy. Trends Cognit. Sci. 18, 163–166. 10.1016/j.tics.2013.12.011 24484764 PMC4560165

[B33] Kim H. Choi H. Han S. (2022). The effect of sense of humor and empathy on the interpersonal adaptation. Pers. Individ. Diff. 197:111791. 10.1016/j.paid.2022.111791

[B34] Lee Y. H. (2019). Emotional labor, teacher burnout, and turnover intention in high-school physical education teaching. Eur. Phys. Educ. Rev. 25, 236–253. 10.1177/1356336X17719559

[B35] Mercer S. H. DeRosier M. E. (2008). Teacher preference, peer rejection, and student aggression: a prospective study of transactional influence and independent contributions to emotional adjustment and grades. J. Sch. Psychol. 46, 661–685. 10.1016/j.jsp.2008.06.006 19083378 PMC2598743

[B36] Meyers S. Rowell K. Wells M. Smith B. C. (2019). Teacher empathy: a model of empathy for teaching for student success. Coll. Teach. 67, 160–168. 10.1080/87567555.2019.1579699

[B37] Naring G. Vlerick P. Van de Ven B. (2012). Emotion work and emotional exhaustion in teachers: the job and individual perspective. Educ. Stud. 38, 63–72. 10.1080/03055698.2011.567026

[B38] O'Brien R. M. (2007). A caution regarding rules of thumb for variance inflation factors. Qual. Quant. 41, 673–690. 10.1007/s11135-006-9018-6

[B39] Okonofua J. A. Paunesku D. Walton G. M. (2016). Brief intervention to encourage empathic discipline cuts suspension rates in half among adolescents. Proc. Natl. Acad. Sci. 113, 5221–5226. 10.1073/pnas.1523698113 27114516 PMC4868443

[B40] Osguthorpe R. D. (2008). Ethical visions in education: Philosophies in practice. J. Moral Educ. 37, 133–135.

[B41] Porat R. Halperin E. Tamir M. (2016). What we want is what we get: Group-based emotional preferences and conflict resolution. J. Pers. Soc. Psychol. 110, 167–190. 10.1037/pspa0000043 26785061

[B42] Preston S. D. (2007). “A perception-action model for empathy,” in Empathy in Mental Illness, eds. T. F. D. Farrow, and P. W. R. Woodruff (Cambridge: Cambridge University Press), 428–447.

[B43] Rajendran N. Watt H. M. G. Richardson P. W. (2020). Teacher burnout and turnover intent. Aust. Educ. Res. 47, 477–500. 10.1007/s13384-019-00371-x

[B44] Riess H. Kelley J. M. Bailey R. W. Dunn E. J. Phillips M. (2012). Empathy training for resident physicians: a randomized controlled trial of a neuroscience-informed curriculum. J. Gen. Intern. Med. 27, 1280–1286. 10.1007/s11606-012-2063-z 22549298 PMC3445669

[B45] Saidah B. Louvet E. Pansu P. (2019). Are students who make an effort perceived as successful or just liked by their teachers? Soc. Psychol. Educ. 22, 405–419. 10.1007/s11218-019-09481-x

[B46] Saloviita T. Pakarinen E. (2021). Teacher burnout explained: Teacher-, student-, and organisation-level variables. Teach. Teach. Educ. 97:103221. 10.1016/j.tate.2020.103221

[B47] Sette S. Gasser L. Grütter J. (2020). Links between teachers' liking of students, peer inclusion, and students' academic achievement: a two-wave longitudinal study. J. Youth Adolesc. 49, 747–756. 10.1007/s10964-019-01048-5 31161273

[B48] Singer T. Seymour B. O'Doherty J. P. Stephan K. E. Dolan R. J. Frith C. D.. (2006). Empathic neural responses are modulated by the perceived fairness of others. Nature 439, 466–469. 10.1038/nature04271 16421576 PMC2636868

[B49] Skaalvik E. M. Skaalvik S. (2017). Dimensions of teacher burnout: relations with potential stressors at school. Soc. Psychol. Educ. 20, 775–790. 10.1007/s11218-017-9391-0

[B50] Stojiljković S. Djigić G. Zlatković B. (2012). Empathy and teachers' roles. Procedia Soc. Behav. Sci. 69, 960–966. 10.1016/j.sbspro.2012.12.021

[B51] Tamir M. Bigman Y. E. Rhodes E. Salerno J. Schreier J. (2015). An expectancy-value model of emotion regulation: implications for motivation, emotional experience, and decision making. Emotion 15, 90–103. 10.1037/emo0000021 25198783

[B52] Tuxford L. M. Bradley G. L. (2015). Emotional job demands and emotional exhaustion in teachers. Educ. Psychol. 35, 1006–1024. 10.1080/01443410.2014.912260

[B53] Waajid B. Garner P. W. Owen J. E. (2013). Infusing social emotional learning into the teacher education curriculum. Int. J. Emot. Educ. 5, 31–48.

[B54] Wang X. Yang L. Chen K. Zheng Y. (2024). Understanding teacher emotional exhaustion: exploring the role of teaching motivation, perceived autonomy, and teacher-student relationships. Front. Psychol. 14:1342598. 10.3389/fpsyg.2023.1342598 38259554 PMC10800834

[B55] Wang X. Zhang L. Peng Y. Lu J. Huang Y. Chen W.. (2022). Development and validation of the empathy scale for teachers (EST). Stud. Educ. Eval. 72:101112. 10.1016/j.stueduc.2021.101112

[B56] Wang Y. Qu C. Luo Q. Qu L. Li X. (2014). Like or dislike? Affective preference modulates neural response to others' gains and losses. PLoS ONE 9:e105694. 10.1371/journal.pone.0105694 25171075 PMC4149476

[B57] Watt H. M. G. Butler R. Richardson P. W. (2021). Antecedents and consequences of teachers' goal profiles in Australia and Israel. Learn. Instr. 76:101491. 10.1016/j.learninstruc.2021.101491

[B58] Weisz E. Cikara M. (2021). Strategic regulation of empathy. Trends Cognit. Sci. 25, 213–227. 10.1016/j.tics.2020.12.002 33386247

[B59] Weisz E. Ong D. C. Carlson R. W. Zaki J. (2021). Building empathy through motivation-based interventions. Emotion 21, 990–999. 10.1037/emo0000929 33211508

[B60] Weisz E. Zaki J. (2017). Empathy-building interventions: a review of existing work and suggestions for future directions. Oxford Handb. Compass. Sci. 2017, 205–217. 10.1093/oxfordhb/9780190464684.013.16

[B61] Weisz E. Zaki J. (2018). Motivated empathy: a social neuroscience perspective. Curr. Opin. Psychol. 24, 67–71. 10.1016/j.copsyc.2018.05.005 29966924

[B62] Wink M. N. LaRusso M. D. Smith R. L. (2021). Teacher empathy and students with problem behaviors: examining teachers' perceptions, responses, relationships, and burnout. Psychol. Sch. 58, 1575–1596. 10.1002/pits.22516

[B63] Wróbel M. (2013). Can empathy lead to emotional exhaustion in teachers? The mediating role of emotional labor. Int. J. Occup. Med. Environ. Health 26, 581–592. 10.2478/s13382-013-0123-1 24057262

[B64] Yaghoubi Jami P. Walker D. I. (2022). Exploring situational empathy and intergroup empathy bias among people with two opposing cultural norms: collectivism and individualism. Int. J. Intercult. Relat. 91, 282–296. 10.1016/j.ijintrel.2022.11.002

[B65] Zaki J. (2014). Empathy: a motivated account. Psychol. Bull. 140, 1608–1647. 10.1037/a0037679 25347133

[B66] Zhang Z. (2022). Toward the role of teacher empathy in students' engagement in English language classes. Front. Psychol. 13:880935. 10.3389/fpsyg.2022.880935 35719575 PMC9201024

[B67] Zhao Q. Neumann D. L. Yan C. Djekic S. Shum D. H. K. (2021). Culture, sex, and group-bias in trait and state empathy. Front. Psychol. 12:561930. 10.3389/fpsyg.2021.561930 33995162 PMC8113867

[B68] Zhu J. Wang X. Q. He X. Hu Y. Y. Li F. Liu M. F.. (2019). Affective and cognitive empathy in pre-teachers with strong or weak professional identity: an ERP study. Front. Hum. Neurosci. 13:175. 10.3389/fnhum.2019.00175 31213999 PMC6555257

